# Experimental study under thermal shock environment to investigate effect of welding width on properties of ultrasonically welded joints of multiple copper cables

**DOI:** 10.1038/s41598-024-73758-1

**Published:** 2024-10-08

**Authors:** Yongqi Zhang, Zeshan Abbas, Lun Zhao, Zhonghua Shen, Liya Li, Jianxiong Su, Saad Saleem Khan, Stephen Larkin

**Affiliations:** 1 Institute of Ultrasonic Technology, Shenzhen Polytechnic University, Shenzhen, 518055 China; 2 School of Mechanical and Control Engineering, Guilin University of Technology, Guilin , 541004 Guangxi China; 3 Faculty of Intelligent Manufacturing Engineering, Guizhou Industry Polytechnic College, Guiyang, 551400 China; 4 United Arab Emirates University, Al-ain, 15551 United Arab Emirates; 5 Africa New Energies Ltd, London, UK

**Keywords:** Ultrasonic welding, Multiple copper cables, Mechanical strength, Microstructure analysis, Thermal shock test, Engineering, Mechanical engineering

## Abstract

Based on the ultrasonic welding technology, this study uses three different welding widths to weld copper cables with different specifications. The influence of welding width on the mechanical properties and microstructure of each group of welded joints was systematically studied for the first time. The thermal shock test was carried out for each group of welded joints under optimum welding width to simulate the influence of severe temperature change environment on joint performance. It is found that the cross-sectional area of joint is 20 mm^2^ and optimal welding width of joint composed of two and three cables is 7 mm. The optimal welding temperature of the joint composed of four cables is 5 mm. Under the optimal welding width, the average shear strength of two-cable joint reaches 309.4 N. The four-cable joint is only 232.2 N. Moreover, the welding strength weakens significantly as the number of cables and the peak temperature decreases. The high temperature of bonding interface is the key factor to form a good weld. The peak temperature during welding is negatively correlated with the porosity of joint and positively correlated with peeling strength of joint. In addition, the morphology of ultrasonically welded joints has changed obviously after thermal shock test. With the participation of oxygen, the surface of welded joint is gray and bright brass, while the interior of joint is purple due to lack of oxygen. Moreover, the phenomenon of atomic diffusion and thermal expansion generates joints which were initially in a mechanically interlocked form and welding interface of the metallurgical bond under the action of high temperature. So the maximum joint peel strength is slightly improved.

## Introduction

With the development of new energy vehicles, the application of wire harness connection in automobiles has become a very important field^1–3^. The crimping technology has been widely used in wire harness connection because of its low cost and high efficiency. However, there is still incomplete contact of wires after crimping and the contact resistance increases in the long-term operation process. The connector is prone to heat, which leads to increased power loss and reduced mechanical strength of welds. This further affects the circuit safety^4–6^. Because the traditional welding technology can’t meet the development needs, ultrasonic welding technology gradually replaces pressure welding and becomes a more stable and safer connection method. It is used for manufacturing conductive parts such as batteries, electronic devices, wire harness terminals and wire connections in electric vehicles, transformers, medical care, aerospace and other fields etc^7–9^. The ultrasonic wire harness welding uses ultrasonic energy to connect two metal wire harnesses together. Its core component is the ultrasonic generator, which generates high-frequency vibration energy and converts mechanical vibration into ultrasonic vibration through the transducer. When ultrasonic vibration acts on the metal wire harness, the oxides and impurities on the metal surface are quickly removed to form a clean metal surface. At the same time, the heat generated by ultrasonic vibration makes the metal surface slightly deformed. Thus, it is realized by welding^10,11^.

The resistivity of copper cable is much lower than that of aluminum cable with the same diameter, and copper cable has the advantages of oxidation resistance and corrosion resistance, so most cars use copper cable^12^. In application, copper cable joints need to bear large current and violent movement, and work in different climates. High quality joints have strong resistance to temperature fluctuation, vibration, moisture and mechanical stress and can ensure their service life and functionality even under poor conditions, greatly improving safety. Therefore, the application market of ultrasonic welding copper cable joints lies in the field of new energy vehicles and the stability of its line joints is very important.

In practical application, the outside temperature of cars in the cold environment in north can be reduced to -40℃ as a minimum. However, the outside temperature is high in tropical and subtropical areas, and the resistance heat generated when a car is quickly charged cannot be cooled quickly. The temperature borne by the car conductive line can reach about 100℃. Therefore, it is particularly important to test and analyze the performance of ultrasonically welded cable joints in extreme temperature environment. A large number of researchers have studied the temperature rise at welding interface during ultrasonic welding of metals^13–15^. The increase of temperature reduces the yield strength of workpiece materials which is beneficial to breakage and plastic deformation of oxide film on the surface of workpiece and plays an important role in formation of joint. Chen et al.^16^ performed a thermodynamic analysis. The heat generation and interface temperature rise were determined by the joint action of friction heat dissipation and plastic deformation heat. The heat generated by plastic deformation accounted for nearly one third of the total calorific value. The highest temperature in the welding process did not reach the melting point of the material, indicating that USW is a solid-state welding process. Zhang and Li^17^ also conducted the similar study and acquired almost the same conclusion after receiving analysis. Siddiq and Sayed^18^ combined the acoustic and thermal softening effects in deformation process, and studied the changes of friction work at the welding interface under different welding amplitudes and different welding pressures.

In recent years, researchers have carried out some research on the microstructure of welding interface in order to understand the mechanisms of ultrasonic welding. Lu et al.^19^ analyzed the grain boundary types and texture evolution during ultrasonic welding. After ultrasonic welding, the material undergoes severe plastic deformation. And continuous < 111 > texture and a large number of large-angle grain boundaries (LAGBs) composed of high-density dislocations and substructures appear at the welding joint surface. When friction heat and deformation heat provide enough energy for recrystallization, dislocation and substructure nucleate and grow into equiaxed grains. Liu^20^, Ma^21^, Fujii^22^ and others found that when Cu plate and Al plate were welded, the grain morphology and size of copper changed little, while the grain of aluminum alloy changed from slender to equiaxed. The texture strength of copper and aluminum alloy initially decreased under the action of ultrasonic softening and then hardened and increased under the action of shear force. Katibi et al.^23^ confirmed the mechanism of dynamic recrystallization and interdiffusion at the thin interface boundary between metal pairs and found that the interface has high thermal stability and strength.

In this paper, joints with the same weld width and total cross-sectional area and different cable combinations are studied for the first time. The influence of joint geometry and cable numbers on the performance of harness joints is analyzed and clarified. EV4, EV6 and EV10 copper cables are combined into three groups and all are 20 mm^2^. The ultrasonic welding is carried out under different welding widths. T-peel test was used to detect the strength of joint to obtain the maximum peeling force. The porosity of the joint was calculated by algorithm and the connection mechanism of joint was discussed in connection with the welding temperature. The micro-morphology of joint formation and joint fracture was observed and the microstructure of welding zone was analyzed in detail. In addition, the thermal shock test was carried out for each group of welded joints under the optimum welding width. Also, the influence of severe temperature change environment on the joint performance was simulated through thermal shock test box. For the first time, the performance of ultrasonically welded cable joints in extreme temperature environments is tested and analyzed in depth.

## Materials and methods

**Fig. 1:**
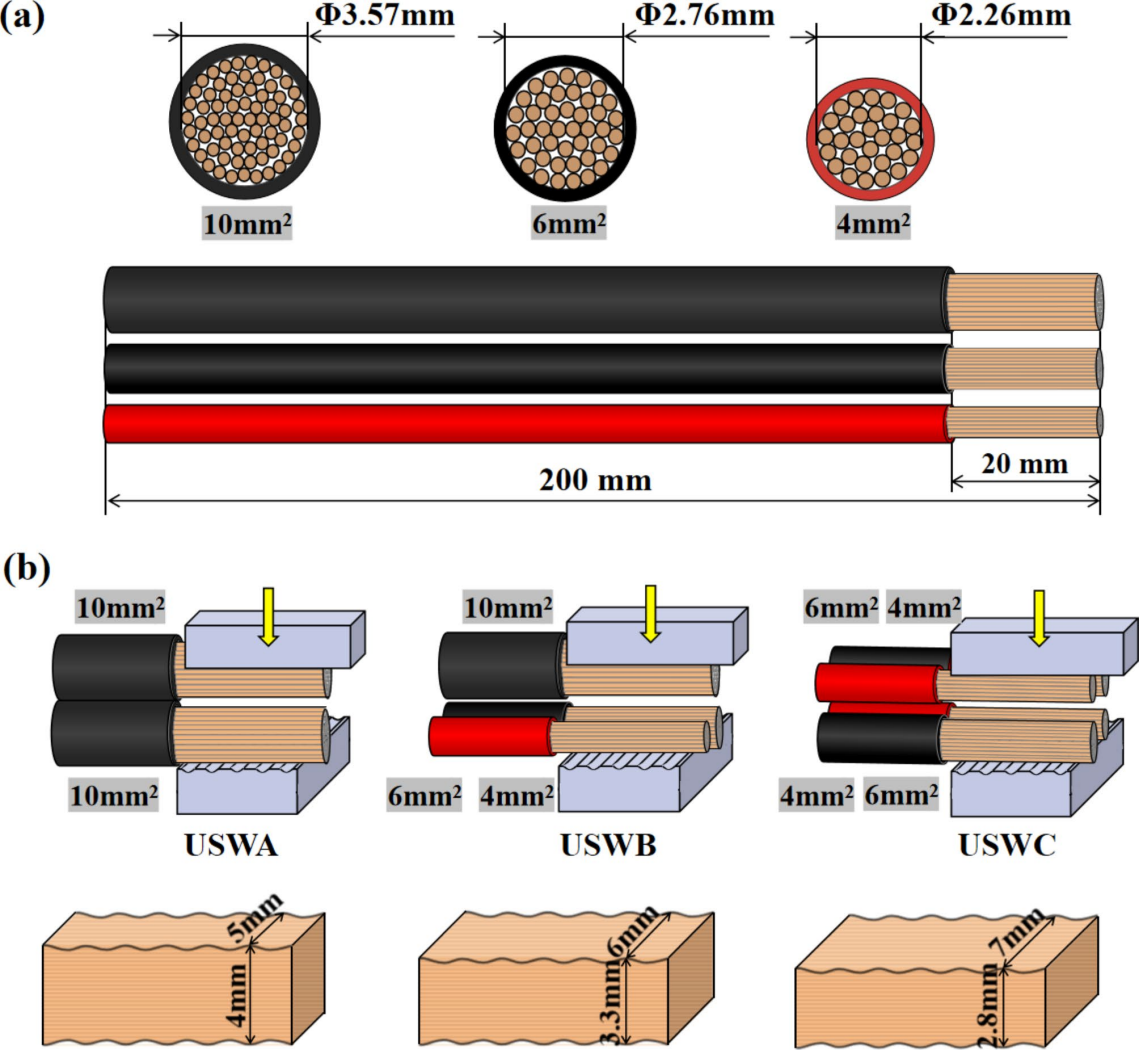
Welding materials and sample preparation diagram (**a**) selected materials and (**b**) process steps.

Three kinds of EV cables with a length of 200 mm are selected for ultrasonic welding with cross-sectional areas of 4 mm^2^, 6 mm^2^ and 10 mm^2^ respectively which are composed of 80, 120 and 200 oxygen-free pure copper wire cores with a diameter of 0.25 mm. The surface PVC insulating material is removed to expose the 20 mm copper core as shown in Fig. 1(a). HMS-X00 ultrasonic beam welding machine was used in the experiment with the working frequency of 20KH and the equipment power of 3000 W. In this study, welding parameters are selected according to the material, thickness and shape in welding requirements of weldments. Its main purpose is to unify welding parameters and facilitate the study of welded joints with different combinations. The welding pressure is 0.55 MPa, the vibration amplitude is 100%, and the welding time is 1600ms. The total cross-sectional area of the ultrasonic welded joint before welding is 20 mm^2^, and three welding combinations are set, namely USWA (ultrasonic welding of two 10 mm^2^ cables), USWB (ultrasonic welding of 4 mm^2^, 6 mm^2^ and 10 mm^2^ cables) and USWC (ultrasonic welding of two 4 mm^2^ and two 6 mm^2^ cables.) as shown in Fig. 1(b). 20 mm² wires are widely used in high current transmission cases. For example, the wires must carry a large current load and long-term operating pressure in the electric vehicle charging station. Therefore, the stability and safety of power transmission can be ensured by choosing wires with a large cross-sectional area. In addition, the welding widths of the three welding combinations are set to 5 mm, 6 mm and 7 mm respectively. The influence of connector geometry and cable number on harness connector performance is analyzed. The joint thickness is different at different weld widths. The geometry of the welded joint is different. Under the same welding parameters, the joint geometry directly affects the transmission and concentration of ultrasonic energy at welding interface. Welding widths of 5 mm, 6 mm and 7 mm are the best choices in experiments and practical applications comparing the joint strength at different widths. Before the welding head works, the left and right parts of the cable are adjusted to positions corresponding to the welding width which limits the lateral movement of the cable and reduces the possibility of wire leakage. Then, the pressing block above the cable presses and goes down to fix the cable. The ultrasonic generator converts the current into high-frequency electric energy and the high-frequency electric energy is converted into mechanical motion with the same frequency again through the transducer. The mechanical motion is transmitted to the welding head through a horn device. The welding head transfers the received vibration energy to the part of cable to be welded, where the vibration energy is converted into heat energy by friction and the parts to be welded are combined^24^.

**Fig. 2:**
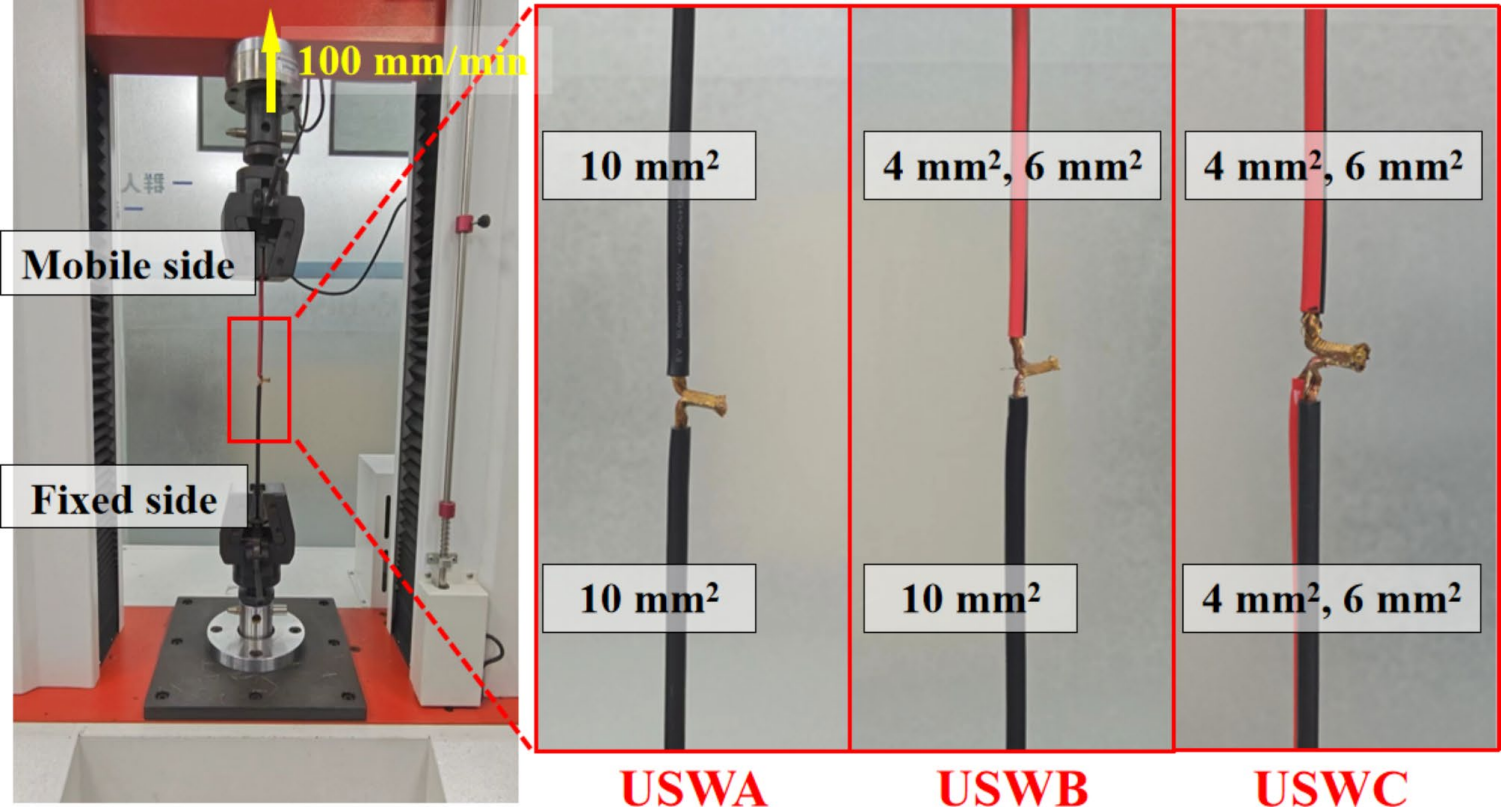
Tensile machine and samples stretching diagram.

**Fig. 3:**
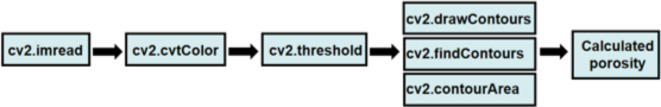
Flow chart of OpenCV library function.

The T-peel test was carried out on welded sample by using Lyxran computer servo material testing machine and the interface connection strength was analyzed experimentally. According to USCAR-45 standard, the stretching speed was set to 100 mm/min and each group was tested for 10 times. During stripping, the cables with a total cross-sectional area of 10 mm^2^ are respectively clamped at upper and lower ends as shown in Fig. 2. According to the standard, it is qualified that the peeling force of the joint with a total cross-sectional area of 20 mm^2^ exceeds 160 N. The middle part of the joint is cut along the welding trace direction by wire cutting, and then polished with 300-mesh, 600-mesh, 1,000-mesh, 1,500-mesh and 2,000-mesh sandpaper in turn, followed by ultrasonic cleaning. The images of welded joint sections are taken by optical microscope, and the porosity of each group of joint sections is calculated by python software. Figure 3 shows the key OpenCV library functions. In this study, the porosity refers to percentage of pore area in the middle section of the joint to total area of the joint section. The temperature measured by K-type thermocouple is related to the joint pore, so as to analyze the interface molding quality. The JSM-IT800 backscattering scanning electron microscope (SEM) was used to observe and analyze the microstructure and failure morphology of the joint section.

**Fig. 4:**
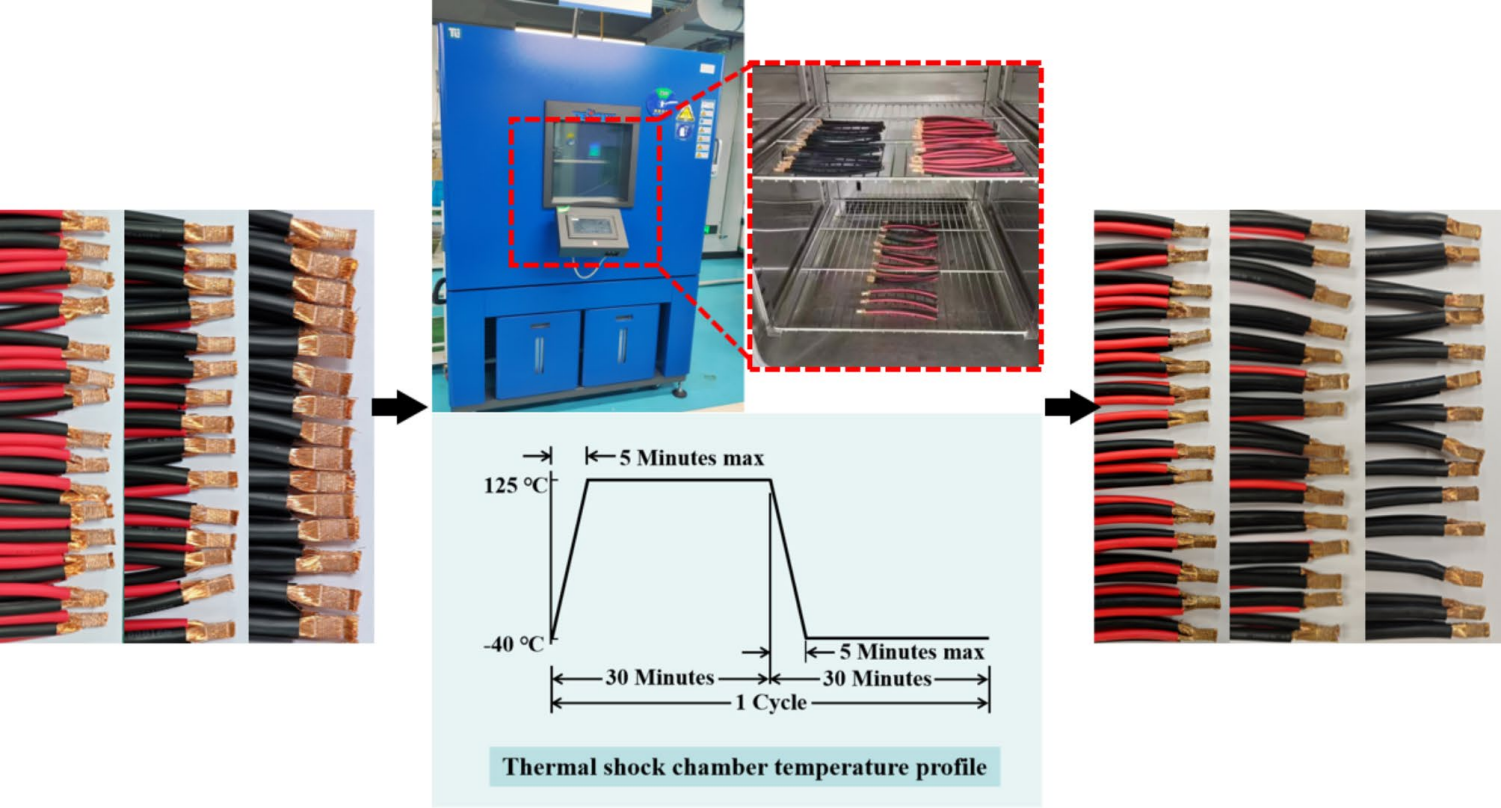
Flow chart of thermal shock test.

After determining the optimal welding width of each group of experiments, TS-T type cold and hot impact tester was used to simulate the environmental adjustment process of alternating high and low temperature environment. The thermal impact test was carried out according to SCE/USCAR-45 standard. The air chamber control was set to temperature and residence time with the highest temperature set to 125℃. The air chamber control was set on temperature and residence time along with the highest temperature set at 125℃. The lowest temperature is set to -40℃ and the number of cycles is set to 72 times. The test program is initiated and samples are removed when the test program ends with the 72 cycle as shown in Fig. 4. Rapid transfer between the two environments tests the ability of welded joint to withstand drastic temperature changes in use of automotive vehicles. After thermal shock test, 10 joints in each group were peeled off. The changes of mechanical properties before and after the test were analyzed and the fracture morphology was observed. CXT2516 resistance test was used to analyze the resistance changes before and after thermal shock test in which two cables with cross-sectional area of 10 mm^2^ were selected for USWA group. The four cables with cross-sectional area of 4 mm^2^ and 6 mm^2^ were selected for USWB group and two cables with cross-sectional area of 4 mm^2^ were selected for USWC group.

## Results and discussion

### Mechanical properties analysis of joint

**Fig. 5:**
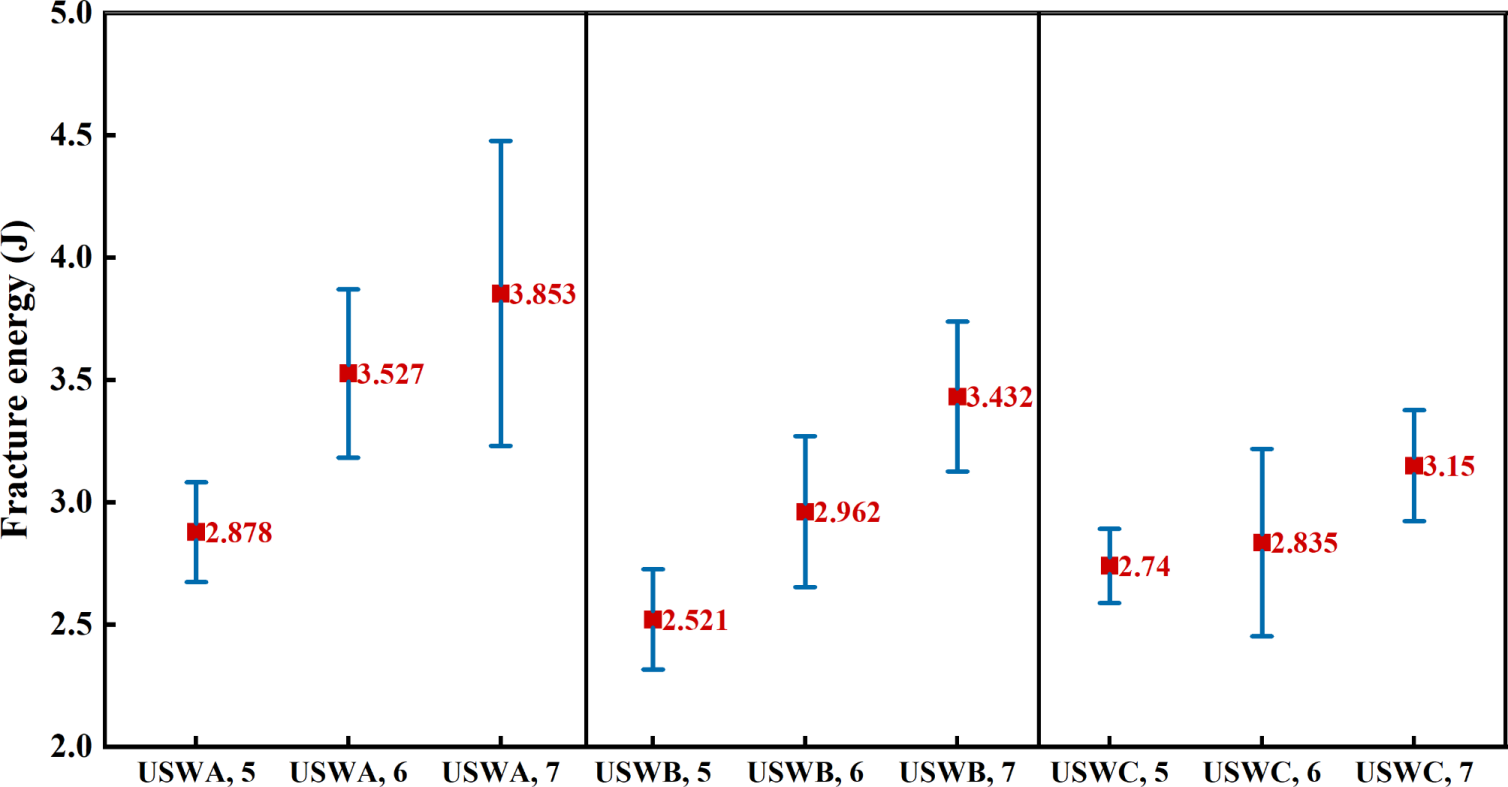
Failure energy of different welding widths of each group of joints.

Figure 5 shows the average and standard deviation of energy required for complete failure after 10 peeling tests of ultrasonic welded joints under different welding widths by three welding combinations. The failure energy is calculated from the area under load-displacement curve. It is found that the failure energy of three groups of welded joints increases with the increase of welding width. It is worth noting that the standard deviation of failure energy at this time is large, while the standard deviation of maximum peeling force is small. It indicates that the dispersion degree of failure energy is high due to the large displacement difference during peeling. The failure energy of welded joints with 7 mm weld width in the USWA group increased by 34% and 9%, respectively, compared to those with 5 mm and 6 mm weld width. The numbers of joints in the USWB group are increased by 36% and 15% and number of joints in the USWC group increased by 15% and 11%. This is because of the increase of welding width. The contact area between cable and welding head is larger and the received vibration energy is transmitted to the part of cable to be welded more effectively. Moreover, it takes more energy for the fracture of welding interface to spread to the whole weld due to increase of peeling joint area. Therefore, it can be judged that the welding width of joint is positively correlated with the failure energy.

**Fig. 6:**
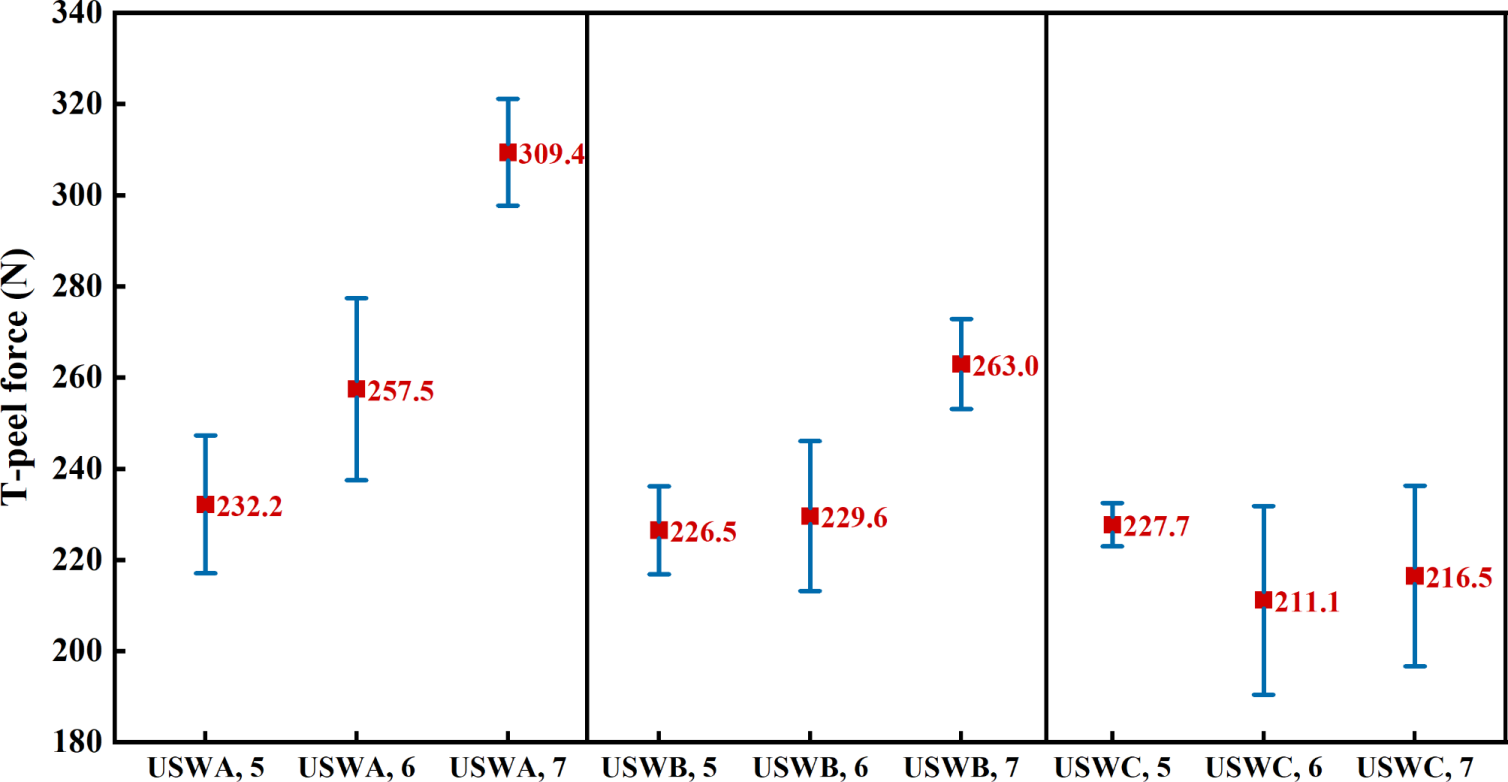
Maximum T-shaped peeling force of different welding widths of joints in each group.

Figure 6 shows the average and standard deviation of maximum peeling force when the ultrasonic joint fails completely after 10 peeling tests of joint under different welding widths by three welding combinations. In USWA group, the maximum peeling force of welded joints with welding width of 7 mm is increased by 33% and 20% compared with those with welding width of 5 mm and 6 mm respectively. The welding width of USWB group is 7 mm and the maximum peeling force increases by 16% and 15% respectively. Increasing the welding width will inevitably lead to a significant reduction in the thickness of the welded joints because the total number of cables is unchanged before welding. The contact area between the welding head and joint is larger under conditions of high width and low thickness. There are more copper wires in direct contact with the welding head. These copper wires first change from the “stick-on” state to the soldered state. At the same time, small thermal resistance and relatively high energy transfer efficiency due to the small thickness of the weld. The welding energy can be quickly transferred to the welding interface during the transfer process and a large amount of energy kinetic energy is converted into bond energy between the metal wires. So, the top and bottom surfaces of the joint can be fully combined. However, there are fewer copper wires in direct contact with the welding head in the case of low width and high thickness. The vibration friction propagates upward along the welding head and amplitude near the bottom is significantly reduced. Therefore, increasing the weld thickness reduces the rate of interface formation during tight bonding.

When the welding width of USWC group is 5 mm, the failure energy of joint is lower than that of the joints with welding widths of 6 mm and 7 mm. However, the maximum peeling force is slightly higher than that of the two groups. Moreover, the failure energy and maximum peeling force are relatively stable at this welding width. This is because in the process of peeling, the joint with welding width of 5 mm only produces a small displacement. Herein, when the joint width is small, the area of the joint with strong welding is also small. The fracture spreads to the whole weld quickly, resulting in the whole joint detachment. The displacement is produced by the material of failure displacement in process of fracture or failure under the action of external force. It reflects the degree of deformation in welded joint from the beginning of the stress to final fracture which is closely related to the mechanical properties of material. Materials with good strength can absorb more energy before fracture because they can undergo large plastic deformations before fracture. When the welding width is 7 mm, the contact area between the joint and welding head is larger and the welding energy acts better on the cable. Therefore, the joint gains better resistance and energy required for failure increases. In addition, the smaller welding width restricts the lateral movement of cables because the USWC group joint is composed of four cables and the welding quality is stable.

It is worth noting that under the condition of welding width of 5 mm, the mechanical properties of three groups of cables with different numbers of cables have hardly changed. This is because the joint thickness is higher when the welding width is smaller. Likewise, the plastic deformation of the joint under welding pressure is smaller than other welding widths. The joint is affected by the amplitude during welding and the copper wire of joint moves towards a uniform area. The smaller welding width makes the copper wire realize a certain degree of homogenization in a shorter time. There is little difference in tiny defects of welded joints with uniform distribution of copper wires. So, the mechanical properties are almost unchanged.

The worst mechanical properties of the joint are obtained by using more cables when the total flatness and width of weld remain unchanged before welding. This is because the copper wires contained in a single cable are often gathered under the influence of insulation rubber of the joint. During ultrasonic welding, the copper wire is first plastically deformed under the influence of welding pressure provided by the welding head, and then it is connected by solid phase under the action of welding amplitude. Therefore, the USWA copper wire is least constrained by the insulation skin, and can be well dispersed and combined under the influence of amplitude during welding. Hence, the best mechanical properties are achieved. However, the number of cables in USWC group is the largest and the copper wires are most constrained by the insulation. Before welding, the copper wires were collected in the center of insulation, which leads to the most uneven distribution of copper wires in welded joints. The larger welding width leads to the further distribution distance between copper wires. The copper wires need more time to realize the uniform distribution inside the joint when subjected to amplitude. The many copper wires have achieved metallurgical bonding before reaching the ideal uniform position inside the joint due to the influence of welding energy and the binding force of insulating rubber. Therefore, uneven copper wires lead to more tiny defects at the interface of multiple cables. When subjected to peeling force, these tiny defects will become the weak points of the whole welded joint. It will take precedence over other joints and lead to a sharp decline in mechanical properties.

The results show that the best welding width of USWA group and USWB group is 7 mm. The USWC group is 5 mm. The maximum peeling force of the joints is observed in each group under the optimum welding width. The strength of the joints in USWA group is the highest, followed by USWB group and USWC group. This shows that the number of cables has a significant impact on weld strength under the same total welding cross-sectional area. The weld strength is obviously weakened when there are more cables.

**Fig. 7:**
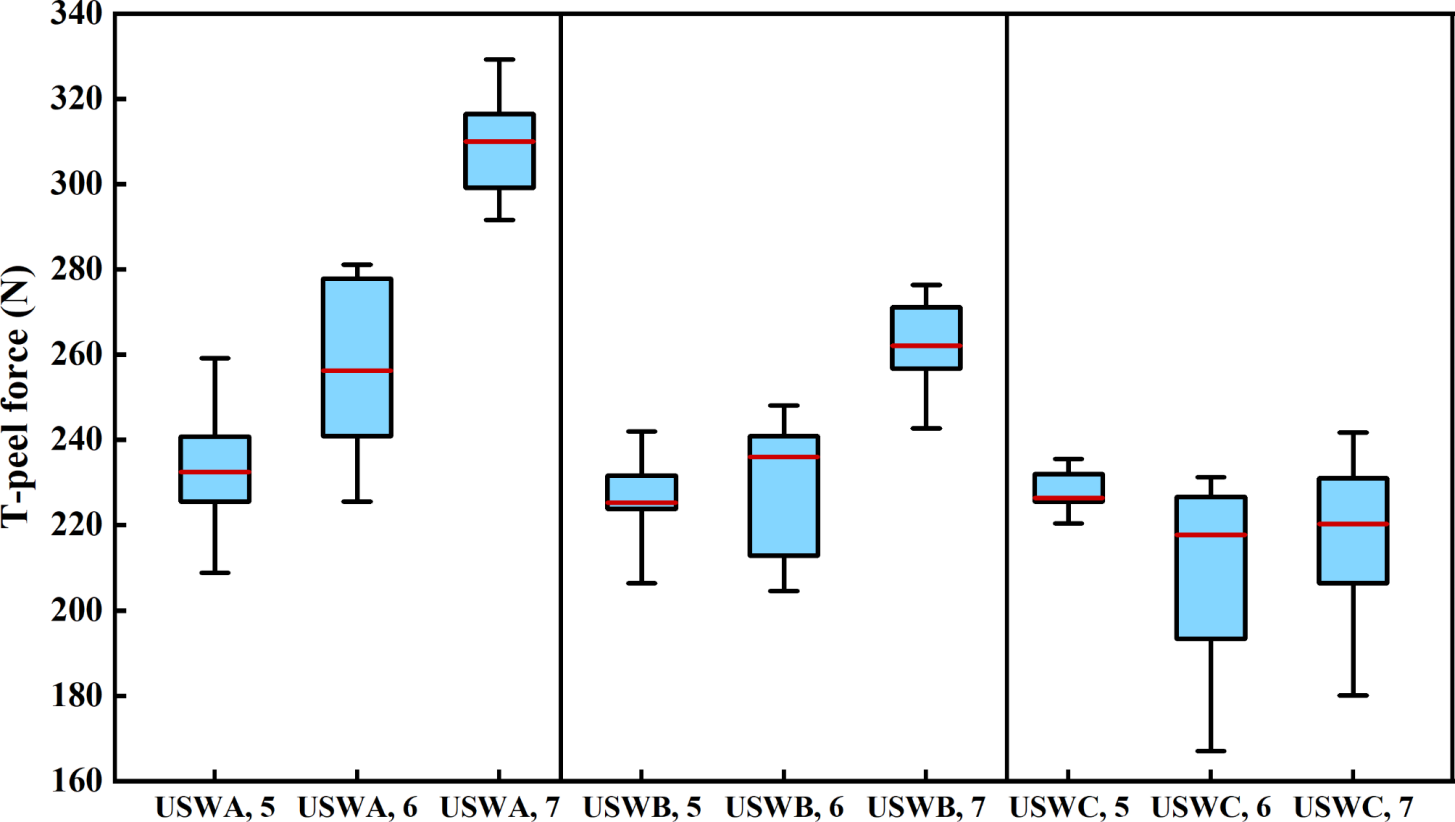
Box diagram of the maximum T-peel force of each group of joints with different welding widths.

Figure 7 shows the box diagram of T-shaped peeling force measured by three welding combinations under different welding widths and the same conclusion is obtained by comparing median values. The median is indicated by the red horizontal line in middle of the box and the upper and lower bottoms of box are the upper quartile (Q3) and lower quartile (Q1) of data respectively, which means that the box contains 50% of data. Therefore, the height of the box reflects wave motion degree of the data to a certain extent. The upper and lower edges represent the maximum and minimum values of this set of data. It is found that the smaller welding width restricts the lateral movement of the cable when the welding width is 5 mm. The formation of keys is more stable and the fluctuation of peeling strength is weak.

### Joint forming analysis

**Fig. 8:**
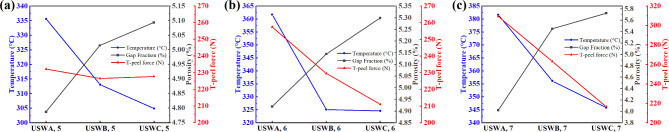
Forming quality analysis (**a**) peak temperature (**b**) joint porosity and (**c**) T-peel force during welding.

**Fig. 9:**
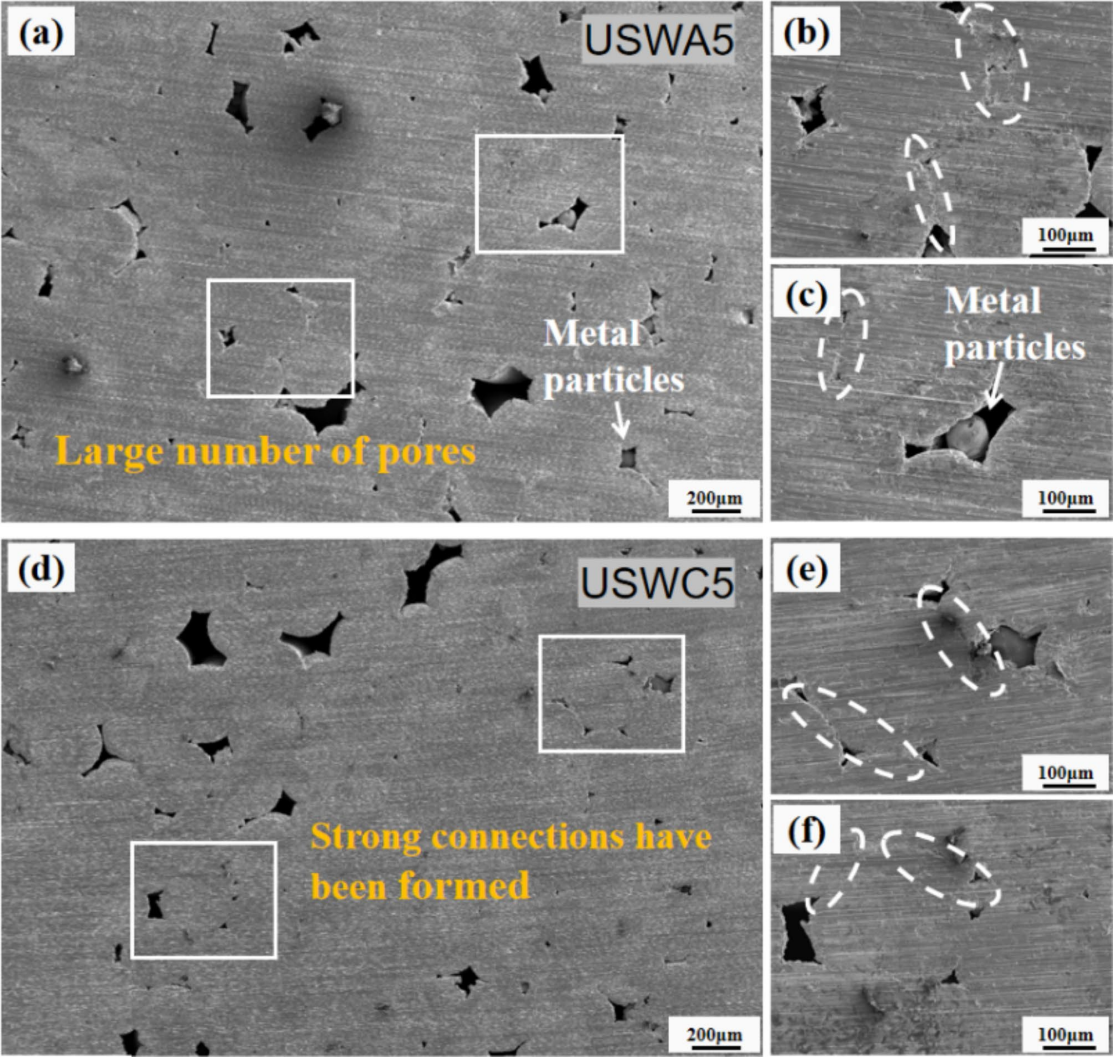
Welding joint section with 5 mm width in USWA5 (**a**, **b**) pores (**c**) metal particles (**d**) surface connection (**e**) void formation and (**f**) solid surface.

Temperature affects the whole deformation process of materials, including dislocation movement within materials, diffusion among members, crystallization process and phase transformation process. In this study, the central area of a welded joint section was selected and the formation morphology observed in each group of joints. The welding temperature, molding quality and peeling strength are related and the relationship among them is studied and analyzed. Figure 8(a-c) shows the peak temperature, joint porosity and T-peel strength of three groups in welded joints under the same welding parameters. When the welding width is 5 mm, the highest welding temperature of USWA group increases by 5.4% compared with USWB group, while the porosity of section decreases by 4.5%. Compared with USWC group, the highest welding temperature of USWB group increased by 2.7%, while the porosity of section decreased by 1.5%. Piezoelectric ceramics drive the sonotrode to generate ultrasonic vibration, and the welding temperature increases rapidly with the increase of welding time. The temperature peaks when the specified welding time is reached during welding. This is useful to reduce the yield strength of a material. Thus, this is used to induce the movement of dislocations to some extent. Dislocation is a local defect of atomic ordering in crystals and its movement has a significant impact on plastic deformation and phase transformation process of materials. At high temperature, the resistance to movement of dislocations decreases. The dislocations are easier to slide and rearrange in the crystal, which helps to improve the microstructure of the materials and the mechanical properties of the welded joints. With the increase of welding width, the welding area of copper cable in direct contact with welding head increases and the thickness decreases. The transmission attenuation of amplitude in welded joint is reduced, and a lot of friction heat and plastic deformation heat are generated between copper wires due to high-frequency vibration^25^. The more amplitude attenuation, the less friction heat and deformation heat are generated, which leads to a downward trend of welding temperature of welded joints. The joint profile of the USWA group was observed and found a large number of pores and dense distribution, which reduces the effective area of solder joint and makes it difficult to create a stable connection between wires as shown in Fig. 9(a). In contrast, the number of small holes in section of the welded joint in USWC group is relatively small and the wires in some areas are closely connected without gaps. So, the welding is stable as shown in Fig. 9(d). It is worth noting that a small number of joints in both groups have only plastic deformation without good welding, and many wire interfaces at the welding site can still be identified. There are metal extrusions and cracks in the pores as shown in Fig. 9(b, c, e and f).

**Fig. 10:**
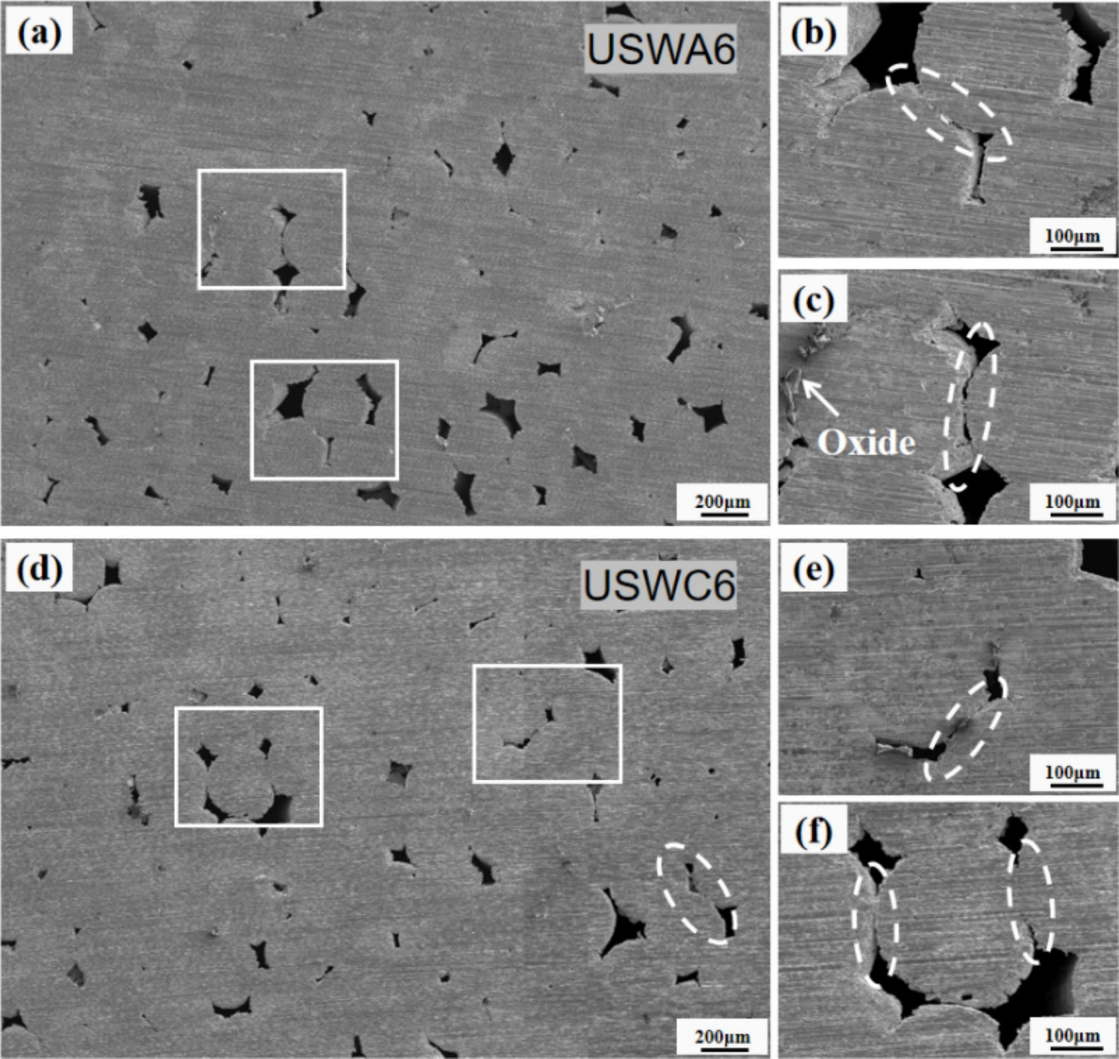
Welding joint section with 6 mm width in USWA6 (**a**, **b**) pores formation (**c**) metal oxide generation (**d**) connection formation (**e**, **f**) void formation with cracks.

The highest welding temperature of USWA group is increased by 11.3% compared to USWB group when the welding width is 6 mm, while the porosity of section is decreased by 4.6% as shown in Fig. 8(b). The maximum temperature difference between USWB group and USWC group is small, while the porosity of section is reduced by 3% and the peeling strength is also improved to some extent. The slight change of peak temperature during welding cannot be ignored for the connection effect between cables. This is consistent with the work of Ding et al.^26^, which described that the cohesive force has little effect on the overall temperature rise. The study observed the joint section of USWA group below this weld width and only plastic deformation still occurs between some cables as shown in Fig. 10 (a, b and c). However, a good connection is not achieved. In contrast, the joint forming quality of USWC group is worse as shown in Fig. 10 (d, e and f). In the initial stage of ultrasonic welding, the clamping force makes the cable surface closely contact and the friction force generated by ultrasonic vibration finely disperses the oxide from weld interface. The original metal surface will approach and form a bond between the atoms^27^. Sometimes, extruded point oxide flakes and metal particles can be observed in the weld as shown in Fig. 9 (a, c) and Fig. 10(c).

**Fig. 11:**
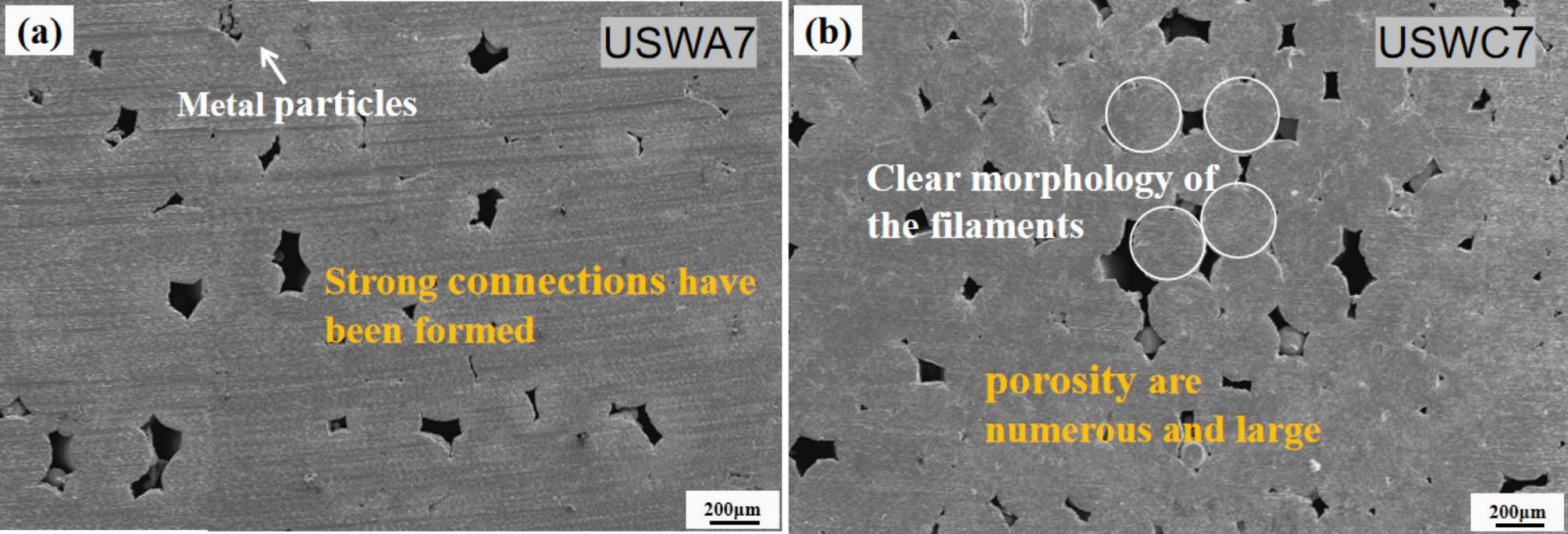
Welding joint section with 7 mm width in USWA7 (**a**) cracks formation along with connection and (**b**) morphology of filaments.

The welding temperature of USWA joint reaches 381.6℃ when the welding width is 7 mm. At this time, it is far below the melting point of copper, which indicates that local melting is impossible during ultrasonic metal welding. However, it has reached the recrystallization temperature of copper. At this time, the material undergoes metallurgical processes such as diffusion, phase transformation and recrystallization, forming solid phase connection. Temperature is caused by the relative motion between cables. It is beneficial to reduce the yield strength of copper cable. It makes the cable locally soft and then the copper cable will undergo serious plastic deformation under the combined action of changing shear force and clamping pressure. Moderate plastic deformation is helpful to accelerate the extrusion of metal into the gap and make materials closer. Thus, it increases the potential position of welding points between copper wires. Besides, the oxide layer is completely destroyed at higher energy and it is easier to form effective micro-bonds. The bonding area is gradually increased. Therefore, a good welding quality is obtained as shown in Fig. 11(a). Micro-bonds at weld interface are the main reason for the formation of the joint. The material continues to move as the welding energy increases and metal gradually flows into the weld interface, resulting in a mechanical interlocking in the joint area^28^. In contrast, the heat generated by welding of USWC group joints is lower than USWA group joints and there are immense pores in the joints. In most areas, a large number of wires have clear shapes and only point connections are formed between wires. Clear filament morphology and some porosity sites were observed in the wire sections as shown in Fig. 11(b).

The results show that the temperature of weld interface is very important to the quality of ultrasonic welded joint. Appropriate weld interface temperature is beneficial to improve weld ability by reducing the yield strength of materials and controlling intermetallic compounds. It further increases the bonding area and reduces formation of pores. Also, the plastic zone size at the crack tip increases correspondingly when the yield limit decreases. The fracture toughness increases and the ability to resist crack propagation increases. When the welding parameters are the same, the peak temperature during welding is inversely related to porosity and positively related to peeling strength. In addition, the forming quality of USWA joint is the best among the three combination methods, followed by USWB joint and finally USWC joint.

### Fracture morphology

SEM was used to observe the fracture morphology under different conditions because the surface of copper wire is curved. The degree of interface contact changes with uneven pressure. So the welding effect in each area is different and bond strength gradually increases from both sides to the middle.

**Fig. 12:**
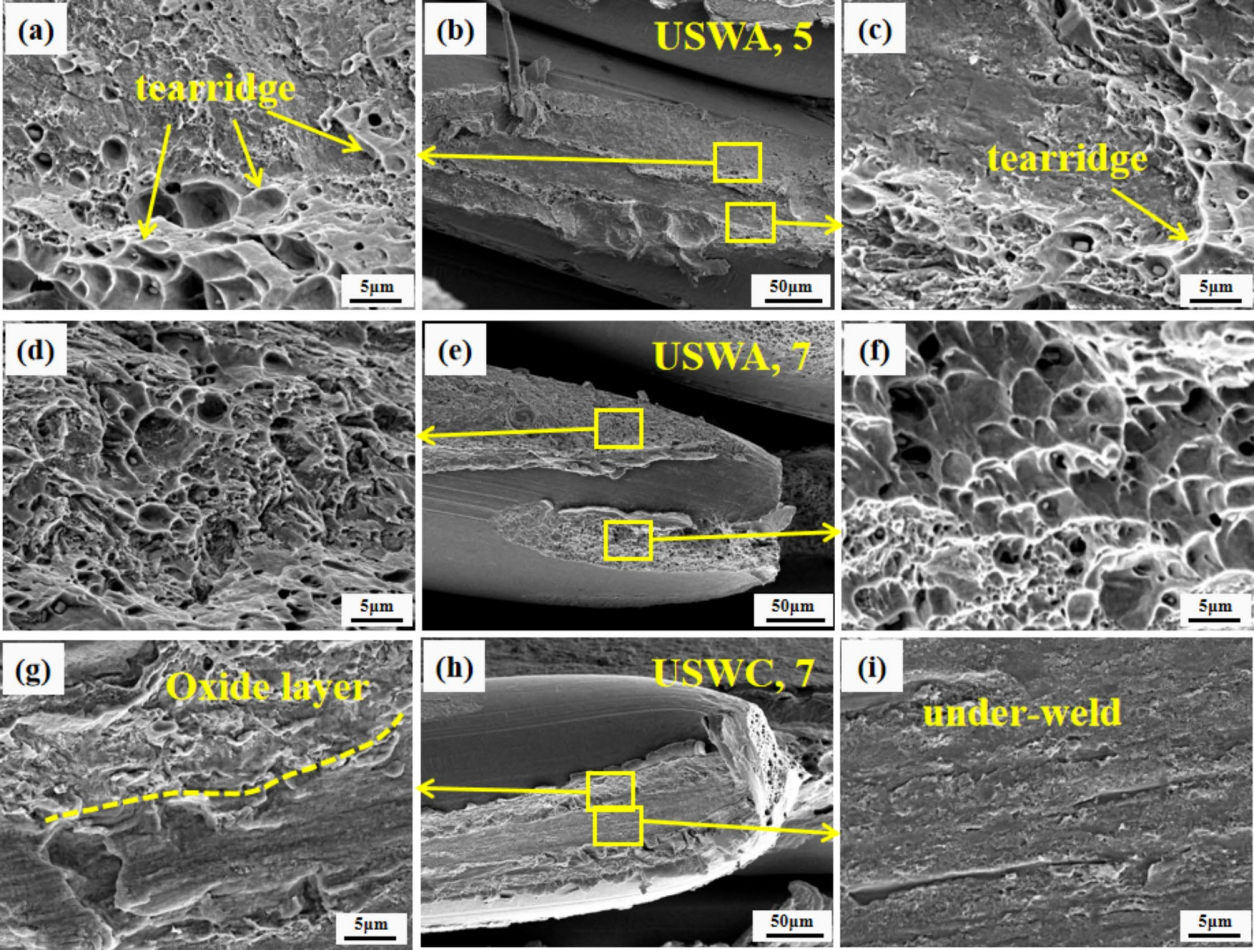
Fracture morphology of joint Welding joint section with 5 mm width in USWA 5 (**a**, **b**, **c**) tear ridges (**d**, **e**, **f**) honey comb morphologies in USWA 7 and (**g**, **h**, **i**) under layer and under weld microscopic morphologies in USWC 7.

As can be seen from Fig. 12(b), the weld periphery of USWA group is relatively flat when the weld width is 5 mm. A few and very small dimples can be observed which do not have a good metallurgical bonding effect. Because of the small width, the bonding surface of the cable is far from a welding head and the ultrasonic energy decays sharply in the process of transmission. The mechanical interlocking connection between copper wires was investigated due to plastic deformation. However, in some areas of copper wire, the oxide layer has ultrasonic softening effect under the action of ultrasonic vibration. A few atoms of bare fresh copper will diffuse at a certain temperature after friction damage and shedding. The connection mode between copper wires has also changed from mechanical embedding to atomic bonding. However, a good metallurgical bonding is formed in the middle bonding area with high pressure and a few large dimples can still be observed. The white ridge around the dimples is called tear ridge as shown in Fig. 12(a, c). The formation of dimple includes the process of micropore formation, growth and connection. Each dimple contains a second phase particle or a broken inclusion or inclusion particle. They are the core of micropore formation^29^.

When the welding width of USWA group is 7 mm, the distance between the welding joint surface and welding head is reduced and the vibration energy is better transmitted to the interface to be welded. Thus, it forms a good metallurgical effect and a large number of dimples with very large size and depth can be observed as shown in Fig. 12(d, e and f). These characteristics show that the material undergoes great plastic deformation, forming excellent metallurgical bonding in the process of T-peel test. The bonding strength of weld is resilient and the crack propagation resistance is improved. There is only minimal internal stress^13^. When the welding interface is peeled off, it can absorb more fracture energy and has higher peeling strength. There are three main stages of good metallurgical bonding. First, under the pressure, the copper cables are plastically deformed after being heated and extruded and the cables are embedded with each other to form mechanical joints. Then, under the action of ultrasonic vibration, the surface of the cable rubs against each other, resulting in the peeling of surface oxide layer and pure Cu and pure Cu contact each other. Finally, with the continuous increase of interface temperature, Cu atoms diffuse with each other to form metallurgical bonding.

When the welding width of USWC group is 7 mm, it is observed that some fracture surfaces are relatively flat as shown in Fig. 12(g, i). During welding, the interface oxide layer was not completely destroyed and only a mechanical joint was formed. In the process of T-shaped peeling, the surface oxide layer falls off under the action of force and some failures are manifested as interfacial adhesion. The nine groups of joints were all accompanied by the fracture of copper wires during the peeling process and all of them showed obvious necking as shown in Fig. 12(e, h). With different metallurgical bonding effects, the dimples with different sizes and depths can be observed at the fracture surface. The results indicate sufficient ductile fracture.

### Thermal shock test analysis

#### Macroscopic morphology analysis

**Fig. 13:**
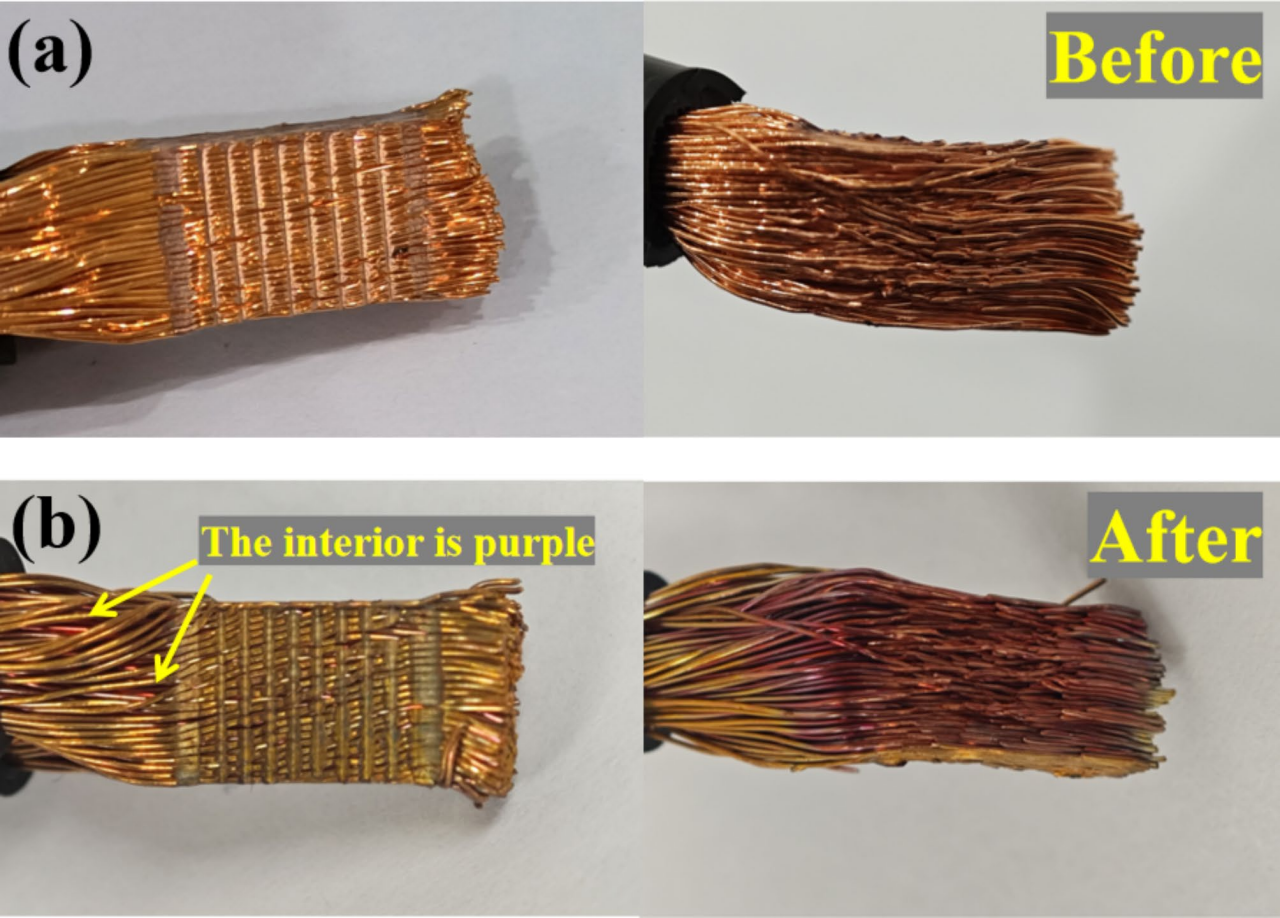
Macromorphology of welded wires (**a**) before and (**b**) after thermal shock test.

The cable is made of pure copper and the color has not changed after ultrasonic welding. The joint appears red and orange. The fracture color obtained by peeling is consistent with the surface color of the joint as shown in Fig. 13(a). After thermal shock, the morphology of ultrasonic welded joints has changed obviously. After thermal shock test, the surface of the joint becomes grayish bright brass, while the inside of the joint is purplish red. The peeling fracture of the joint is purplish red as shown in Fig. 13(b). This is because in the process of thermal shock measurement, the oxidation rate of copper is obviously increased at high temperature and the surface of the joint fully reacts with oxygen. In this case, copper is oxidized at high temperature to form copper oxide. However, there are differences in oxygen content and oxidation products in each region, which makes the color of the joint surface and interior different^30^. In addition, they will enter in the high-energy state when electrons absorb energy. The high-energy electrons jump back to the low-energy state and will release photons and change their colors. The oxygen molecules are adsorbed on the surface of joint and form chemical bonds with copper. Copper oxidation mainly involves three chemical reactions as follows in Eq. (1).

1 $$\begin{aligned} 2Cu+\frac{1}{2}{O_2}&=C{u_2}O \hfill \\ Cu+\frac{1}{2}{O_2}&=CuO \hfill \\ \frac{1}{2}C{u_2}O+\frac{1}{4}{O_2}&=CuO \hfill \\ \end{aligned}$$

#### Mechanical property analysis

**Fig. 14:**
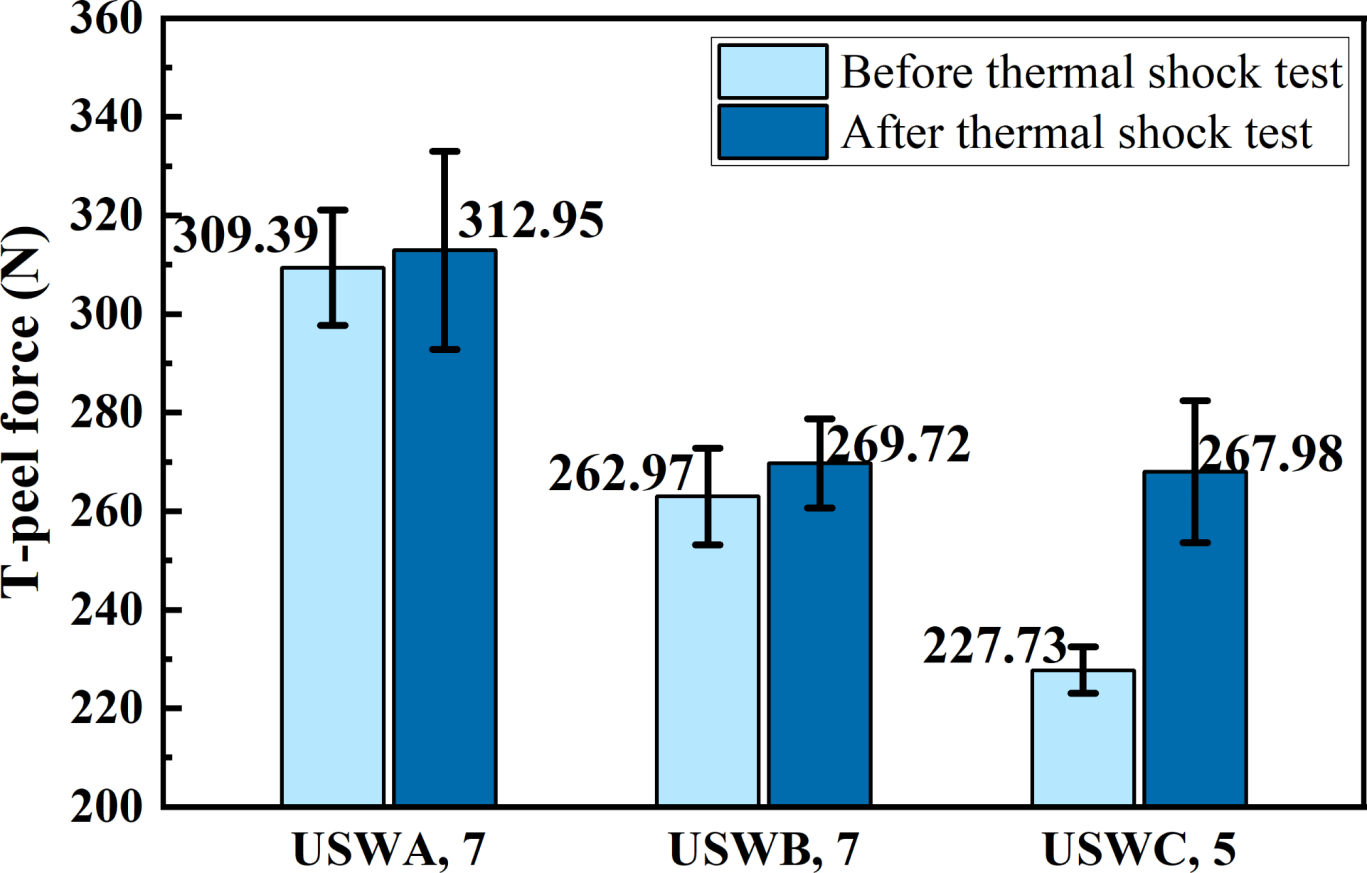
Maximum T-peel force before and after thermal shock test.

It is found that the maximum peeling force of joint is slightly improved after thermal shock test as shown in Fig. 14. Among them, the joint in USWC group is the most significant and the average peeling force of tested joint is increased by 17.7% compared with the original joint. After the ultrasonic welding of USWC group was completed, plastic deformation occurred between some copper wires, but good bonding was not obtained. In the high temperature environment, the thermal movement of atoms breaks the position constraint of crystals and produces transposition and movement which leads to atomic diffusion. In the process of phase transformation and precipitation, copper atoms change the phase structure and composition through diffusion. Moreover, the material expands when the temperature of the joint rises sharply. However, the internal materials in contact with each other limit its free expansion, so the interior of the joint is subjected to compressive stress^31^. Consequently, the originally formed mechanical interlocking joints form the welding interface of metallurgical connection under the action of mutual diffusion between atoms. In the thermal shock experiment, the joint will experience a rapid change in temperature which will lead to redistribution of internal stress in the material. With the increase and decrease of temperature, the material will undergo thermal expansion and contraction which will help to release the original stress and make the joint structure more stable. Thus, it improves the mechanical properties. In addition, under the condition of transition from high temperature to low temperature, the grains of pure copper will produce internal stress due to the difference of thermal expansion coefficient. Furthermore, it may lead to the cracking of grains in a specific direction and form a new grain boundaries and leading to grain refinement. Grain refinement will improve the strength and hardness of materials because fine grains can effectively hinder the movement of dislocations. Likewise, the deformation of grain is limited at low temperature, which leads to the rearrangement and reconstruction of grain boundaries. The formation of more complex grain boundary structure leads to the increase of grain boundary number^32^. Pure copper generally does not undergo phase transformation in the range of -40℃ to 125℃. However, it may induce tiny phase transformation under extreme thermal shock cycle conditions. These changes jointly affect the mechanical properties of welded joints and improve the mechanical properties of joints. Conversely, the stability of the joint is reduced because of the uneven internal changes in joint.

#### Resistance analysis

**Fig. 15:**
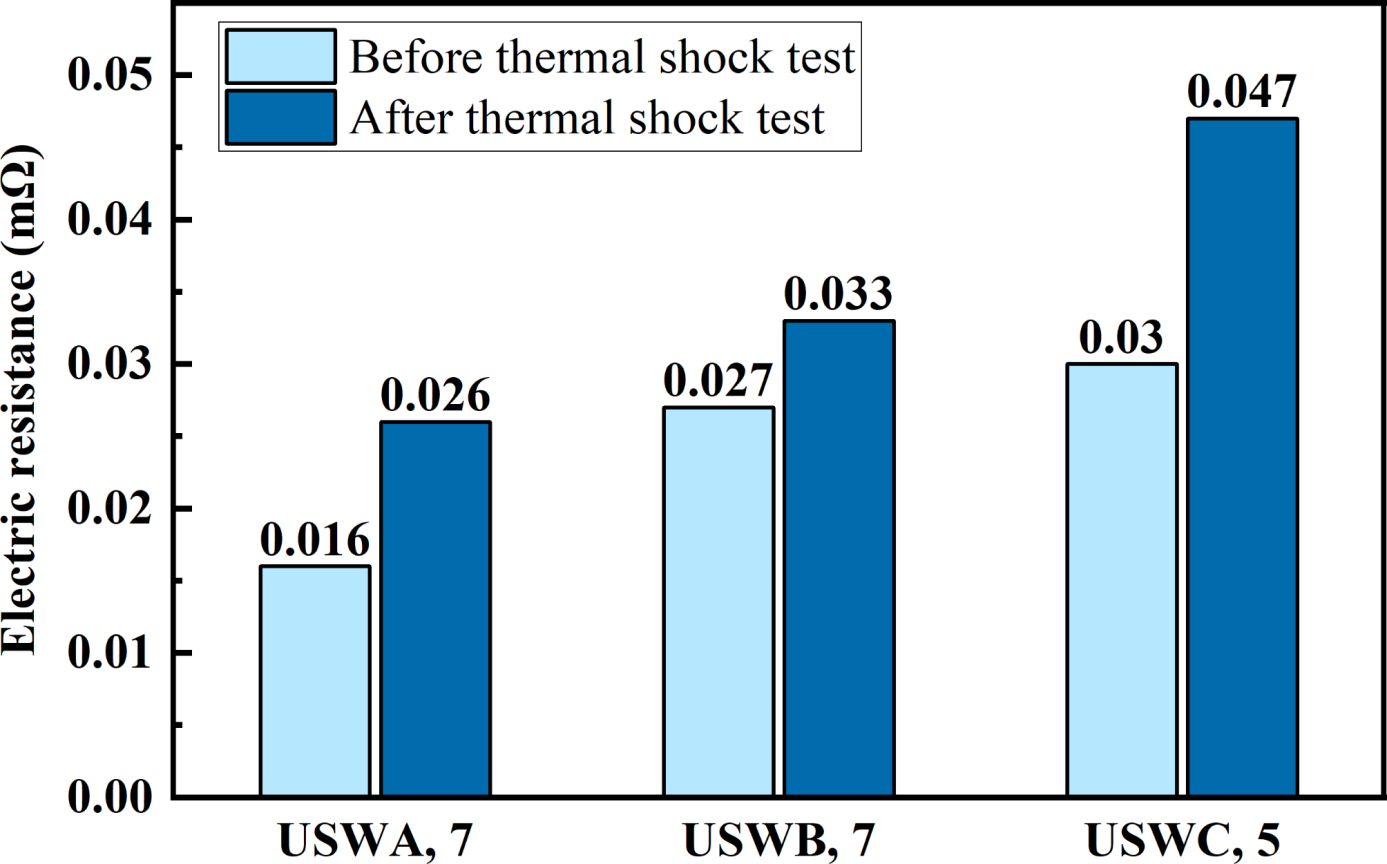
Resistance before and after thermal shock test.

Temperature has a great influence on resistance, which is one of the main reasons for the change of resistivity. The higher joint resistance will generate Joule heat, which will lead to excessive energy loss and reduce power supply capacity and efficiency^33^. The change of temperature will affect the physical characteristics of resistance such as the size, structure and material properties of resistance. After thermal shock test, the resistance of each group of joints increased as shown in Fig. 15. Among the three groups of joints, the change of USWC joint is the biggest and the resistance of the joint after thermal shock test is increased by 56.7% compared with the original joint. Repeatedly raising and lowering the temperature will change the physical structure of the joint slightly and the atoms in the resistive medium will move laterally with the temperature change. The inherent size and structure of the resistive material will change. Thus, it changes the resistance. The resistivity is closely related to the concentration of point defects in metals. The point defects in materials can’t recombine in temperature change, which leads to the existence of supersaturated point defects. The flow of electrons directionally is subjected to unbalanced force, which increases the resistance and resistivity.

In addition, the internal energy of pure copper increases in the high temperature environment and it is easier to absorb oxygen atoms to react. Moreover, the oxidized metal has higher resistance. The gray brass color formed on the copper surface after the impact of extreme temperature changes copper oxide (CuO). Copper oxide is a semiconductor material and its resistivity is between conductor and insulator. However, the copper wire inside the joint turns purple, which is cuprous oxide (Cu_2_O) produced by pure copper joint due to temperature change in oxygen-deficient environment. The copper oxide and cuprous oxide are oxides of copper, and their conductivity is much lower than pure copper. Therefore, when these oxides are formed on the surface or inside the copper wire and will act as a resistance source, resulting in an increase in the resistance of the entire copper wire joint.

#### Fracture morphology

**Fig. 16:**
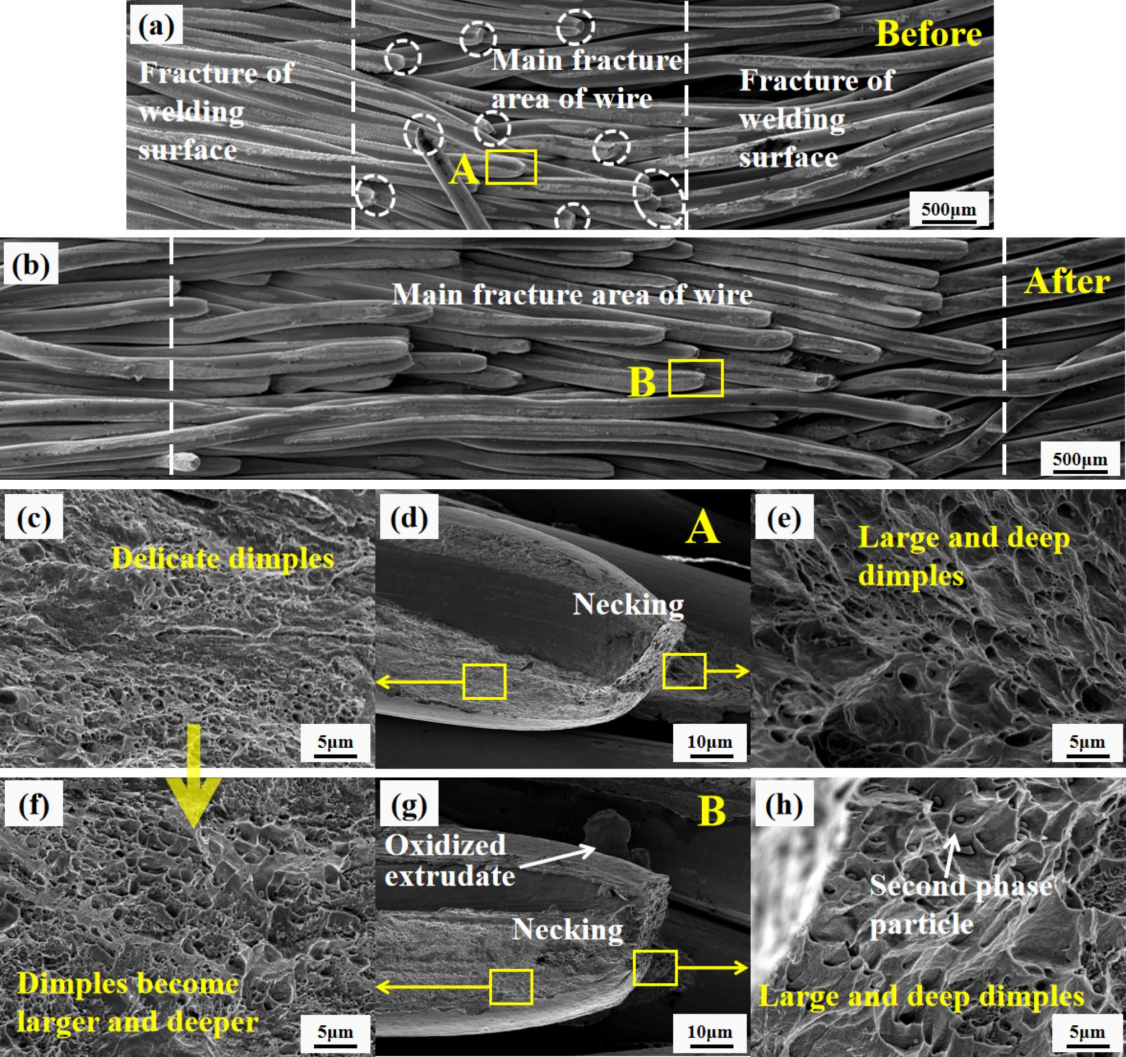
Fracture morphology of USWC group joint before and after thermal shock test (**a**) fracture of welding surface (**b**) main fracture areas on top of wire (**c**) delicate dimples (**d**) necking (**e**) large and deep dimples surface (**f**) honey comb surface (**g**) oxidized and necking surface and (**h**) second phase particles.

The peeling fracture of the original joint of USWC group with welding width of 5 mm before thermal shock test is observed. The welding interface on both sides of the fracture shows failure and wire breakage rarely occurs. Wire breakage is mainly concentrated in the middle area of the joint fracture as shown in Fig. 16(a). This is because the weld strength on both sides of the joint is low and wires are easily separated when subjected to external force. However, the welding quality is better in the middle area with high weld strength. The plastic deformation begins to appear in the weakest part of the wire when the shear resistance between wires is greater than the tensile strength of wire core under tensile stress leading to the sharp necking of local section of the copper wire. At this time, the diameter of the wire is greatly reduced. The load on the wire can bear is rapidly reduced until it breaks as shown in Fig. 16(d, g). There are weld fractures with broken wires and a large number of dimples with large size and deep enough can be observed as shown in Fig. 16(e, h). However, the failure morphology without wire fracture is observed. Further, it is found that the dimple size is obviously small and the depth is shallow as shown in Fig. 16(c, f).

After the thermal shock test, the ultrasonic welded joint is accompanied by a large number of wire fractures in the whole peeling process and the wire fracture area extends from the middle area of joint fracture to the whole joint fracture as shown in Fig. 16(b). This shows that the weld strength on both sides of the joint is obviously improved after thermal shock test and the wires are not easily separated when peeling. Many welding interfaces are no longer mechanically interlocked by plastic deformation of materials. High temperature accelerates the diffusion of atoms and the compressive stress caused by thermal expansion inside the joint evolves into the welding interface of metallurgical connection. By observing the micro-morphology of fracture failure in each area, it is found that the dimple shape produced by peeling after thermal shock test is larger and deeper as shown in Fig. 16(c, f). In addition, the second-phase particles are relatively larger and easier to observe as shown in Fig. 16(h). In the process of joint peeling, the stress rises sharply when the stress is concentrated in a certain area of copper wire, which makes the bonding force between atoms fail. The plastic deformation of a component is different in different parts, so there will be material discontinuity in some parts with large plastic deformation. Moreover, the formed micropores are the major source of fracture failure^34^. The micropores become larger and then merge under the action of peeling force, resulting in dimples. Therefore, the better the joint plasticity is obtained and larger the dimple size.

## EDS analysis

**Fig. 17:**
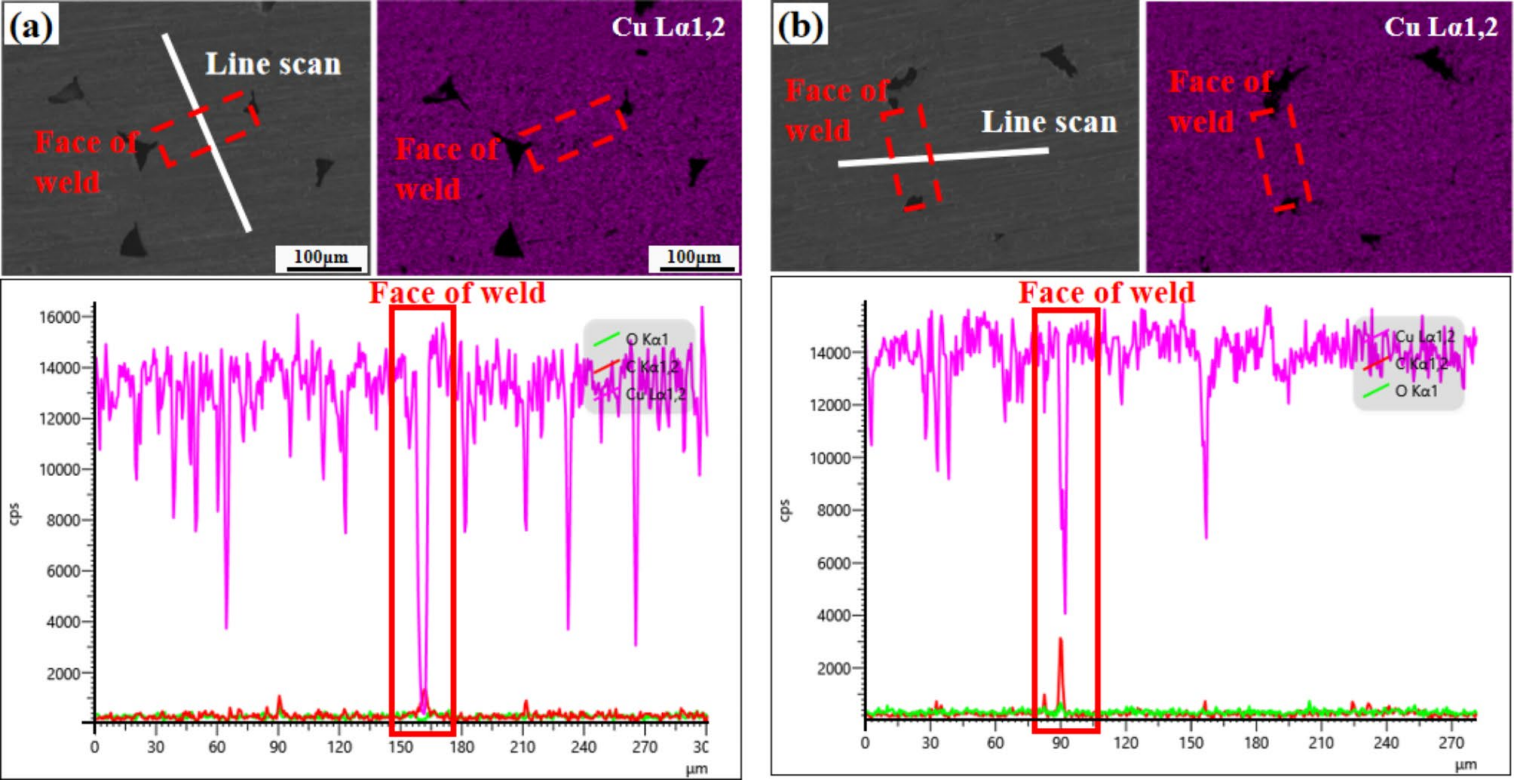
EDS analysis of USWC joint before and after thermal shock test (**a**) line scan before test and (**b**) line scan after test.

Energy Dispersive X-ray Spectroscopy (EDS) analysis was carried out for USWC group joints without thermal shock test and the central position of joint section was selected. The distribution of Cu in different areas was analyzed by surface scanning and line scanning as shown in Fig. 17(a). It is found that at the weld of two copper wires, there is a sharp decrease of Cu in the area of 5 μm and only a very small amount of Cu exists and the eps value is lower than 1000. It shows that the copper wires are mechanically interlocked after plastic deformation of the material in the process of ultrasonic welding and only a few copper atoms are bonded. So, the welding effect is poor and there are extremely fine gaps between the wires. This cannot be observed in the micro-morphology of the joint section. These tiny cracks will become the weak points in the peeling experiment, which will directly lead to the poor stability of welded joint and reduce the mechanical strength of joint.

The same area for EDS analysis of USWC joint was selected after thermal shock test the distribution of Cu in different areas through surface scanning and line scanning was analyzed as shown in Fig. 17(b). However, the Cu element changes in the area where there is only 3 μm in the solder layer of two copper wires, and the eps value in area with the smallest content of element decreases to 4000. In the same area, the content of Cu has been greatly improved compared with the original joint without thermal shock test. Once again, it is proved that at a certain temperature, the mechanical interlocking joints that were originally formed form the welding interface of metallurgical connection under the action of mutual diffusion between atoms. Thus, it improves the mechanical strength of welded joints. The results show that the more Cu content in the weld, the greater the strength of the welded joint.

## Conclusions

In this study, ultrasonic welding technology is carried out and three welding width parameters are used to weld cables of different specifications. The welded joint was studied and analyzed by peeling test, temperature measurement and microstructure. In addition, the thermal shock test was carried out for each group of welded joints with the best width to simulate the influence of alternating high temperature and low temperature environment on welded joints. The following conclusions were drawn.

1. The best welding width of USWA group and USWB group is 7 mm and USWC group is 5 mm.
2. The number of cables has a significant influence on the weld strength when the total welding cross-sectional area is similar. Also, the weld strength is obviously weakened when the number of cables is large. Under the optimum welding width, the average peeling force of USWA joint reaches 309.4 N. However, there are a large number of weld interfaces in USWC group that only undergo plastic deformation without micro-bonds and the average peeling force is 232.2 N.
3. The key factors of forming a good weld joint combined with the high temperature of interface. The peak temperature during welding is negatively correlated with the porosity of joint and positively correlated with the peeling strength of joint.
4. After thermal shock test, the morphology of ultrasonic welded joint changed obviously. The surface of joint produced bright gray brass copper oxide (CuO), while the interior of joint produced purplish red cuprous oxide (Cu_2_O). The peeling strength is slightly improved. However, the electrical conductivity of joint is decreased.
5. T-shaped peeling fracture characterized by dimple indicates that the failure mode ofjoint is ductile fracture. After thermal shock, the fracture area of wire extends from the middle area of joint fracture to whole joint fracture. It was investigated that high temperature accelerates the diffusion of atoms and compressive stress caused by thermal expansion within the joint evolves at weld interface of metallurgical joint. Further, it leads to larger and deeper forms of dimples produced by the peeling effect.
6. The selection of welding width parameters is very important for ultrasonic welding of multiple cables. It directly affects the quality, efficiency and reliability of welding. It is one of the main factors to ensure the strength and durability of welded joints. In addition, the thermal shock test is essential for the study of ultrasonically welded joints. The thermal shock test can verify the temperature change resistance of welded joints, optimize welding process parameters, meet the requirements of specific industries and promote technical development. These entire factors will help to improve the overall level and application effect of ultrasonic welding technology.

## Data Availability

The datasets used and/or analyzed during the current study are available from the corresponding author upon reasonable request.
